# Dual Circularly Polarized Planar Four-Port MIMO Antenna with Wide Axial-Ratio Bandwidth

**DOI:** 10.3390/s20195610

**Published:** 2020-09-30

**Authors:** Sachin Kumar, Gwan Hui Lee, Dong Hwi Kim, Hyun Chul Choi, Kang Wook Kim

**Affiliations:** School of Electronic and Electrical Engineering, Kyungpook National University, Daegu 41566, Korea; gupta.sachin0708@gmail.com (S.K.); gwan6088@knu.ac.kr (G.H.L.); eastsine766@gmail.com (D.H.K.); hcchoi@ee.knu.ac.kr (H.C.C.)

**Keywords:** diversity, dual circularly polarized, isolation, MIMO, planar

## Abstract

A broadband compact-sized planar four-port multiple-input–multiple-output (MIMO) antenna with polarization diversity is presented. The proposed dual circularly polarized (CP) MIMO antenna consists of four G-shaped monopole elements, two of which are left-hand CP and the other two are right-hand CP. A vertical line strip in the G-shaped radiating element acts in balancing the vertical and horizontal electric field components to obtain 90° phase difference between them for circular polarization. Also, an I-shaped strip is incorporated between the ground planes of the G-shaped antenna elements to obtain equal voltage level in the proposed MIMO configuration. The dual circular polarization mechanism of the proposed MIMO/diversity antenna is analysed from the vector current distributions. The impedance bandwidth (S_11_ ≤ –10 dB) of the MIMO antenna is 105.9% (4–13 GHz) and the 3 dB axial ratio bandwidth (ARBW) is 67.7% (4.2–8.5 GHz), which is suitable for C-band applications. The overall size of the MIMO antenna is 70 × 68 × 1.6 mm^3^, and the minimum isolation between the resonating elements is 18 dB. The envelope correlation coefficient is less than 0.25, and the peak gain within the resonating band is 6.4 dBi.

## 1. Introduction

The introduction of spatial diversity in wireless communication systems has improved their performance significantly [1,2]. Multiple-input–multiple-output (MIMO) antenna technology exploits the phenomenon of multipath signal propagation, and employs multiple antennas at transceiving ends to send and receive signals. As compared to single antenna systems, the MIMO arrangement provides high spectral efficiency, high capacity, and reliable communication between the transmitting and receiving terminals [3]. In multi-antenna systems, the coupling among radiating elements reduces the performance of the system and, therefore, various techniques have been explored to increase inter-element isolation [4,5]. The orthogonal placement of the antenna elements in a MIMO configuration was frequently used as one of the efficient decoupling methods but, at the same time, it increases the design complexity [6]. Other decoupling methods used neutralization lines, electromagnetic band-gap (EBG), frequency-selective surface (FSS), and parasitic elements [5]. However, these decoupling techniques employed some additional active/passive elements, which added to the overall footprint of the antenna [7]. Therefore, techniques like polarization/pattern diversity were used to reduce far-field correlation without any increase in the antenna size [8,9]. The polarization diversity not only decreases the inter-element coupling, but also offers superior antenna performance by improving diversity gain and channel capacity [10,11].

Circularly polarized (CP) antennas have been widely used in many modern wireless communication systems due to their immunity to multipath propagation [12,13,14,15]. Moreover, the transmission or reception of signals does not depend on the antenna orientation [16]. Antennas with multiple polarizations provide the benefit of frequency reuse and increased channel capacity. Therefore, dual-polarized antennas with MIMO features have been gaining popularity in recent years. In the last five years, various CP MIMO antenna configurations have been presented for wireless local area network (WLAN), C-band, and satellite applications [17,18,19,20,21,22,23,24,25]. In [17], a dual-port antenna with two resonating elements was proposed for WLAN applications, where the upper square patch with truncated corners showed CP behaviour, while the bottom interdigital-type radiator showed linearly polarized (LP) behaviour. A triple-port MIMO antenna comprised of two LP dipoles and one CP chamfered-edge square patch antenna was presented for WLAN systems [18]. In [19], a dual-CP coplanar waveguide (CPW)-fed square slot antenna with two asymmetric T-shaped feed lines was presented, where inverted-L strips were used in the ground plane for obtaining wide axial ratio bandwidth (ARBW). In [20], a patch antenna with protruded L-shaped and inverted L-shaped strips for producing dual circular polarization was suggested. In [21], a compact MIMO antenna with polarization diversity was reported, where three grounded stubs and an F-shaped defected ground structure (DGS) were used to obtain improved inter-element isolation. A CP MIMO antenna comprising two electromagnetically coupled circular-shaped monopole radiators was presented [22], where inter-element coupling was improved by using a rectangular decoupling element. In [23], a two-port CP MIMO antenna was presented with polarization diversity, where two CPW-fed monopole elements were located opposite each other for obtaining dual circular polarization. A triple-band MIMO antenna consisting of four resonating patches with truncated corners was presented in [24], where large spacing was provided among the resonators to improve isolation. A four-port CP antenna consisting of truncated-corner cross-slot-loaded patches was proposed for 5.8 GHz WLAN band [25], where a two-arm feed mechanism was used to excite the resonating elements. However, the CP MIMO antennas reported in [17,18] could generate only right-hand CP (RHCP) waves. The CP MIMO antennas in [19,21,23,25] offered dual circular polarization but exhibited very small axial ratio and impedance bandwidths. Moreover, in most of the above-reported CP MIMO/diversity antennas, the ground planes of the antenna radiators were not associated to each other and, therefore, these antennas could not be used in practical applications [26].

In this article, the design method of a compact planar dual-CP four-port MIMO antenna is presented. The designed MIMO/diversity antenna consists of four G-shaped monopole elements, which are fed through 50-Ω microstrip lines. The antenna radiates RHCP waves when the elements 1 and 3 are excited, and left-hand CP (LHCP) waves when the elements 2 and 4 are excited. An I-shaped strip is incorporated between the ground planes of the antenna elements, which connects the four ground planes, setting the equal voltage level in the proposed MIMO configuration. In addition, the I-shaped strip increases isolation between the four-elements and helps in restoring wide ARBW of the antenna element.

## 2. Antenna Configuration

### 2.1. Antenna Element Design

**Figure 1:**
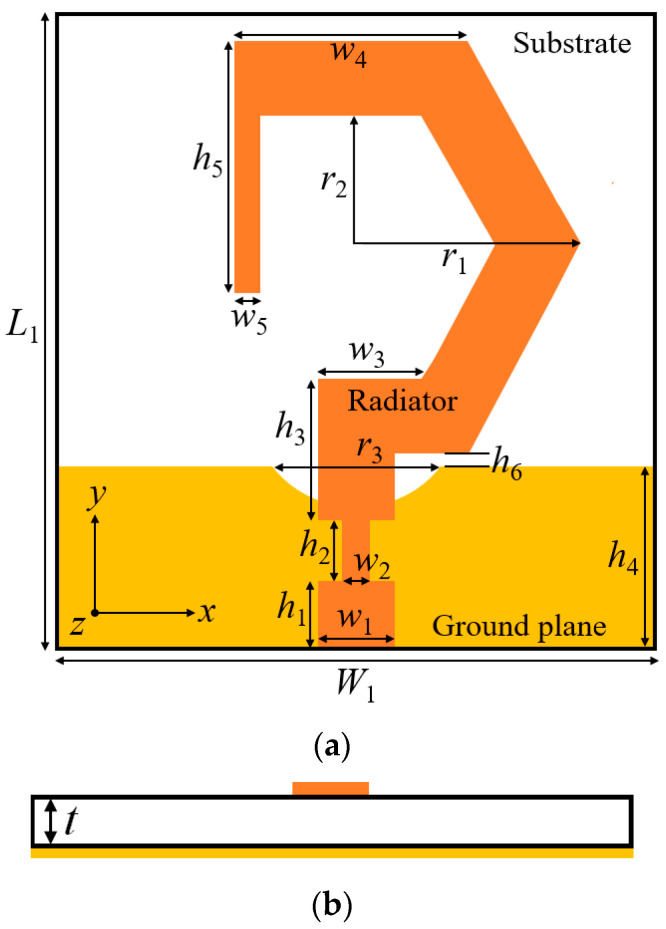
Schematic layout of the proposed circularly polarized (CP) monopole antenna element: (**a**) top view; (**b**) side view.

The top and side views of the monopole antenna, used as a radiating element of the proposed MIMO antenna, are illustrated in Figure 1a,b, respectively. The antenna element is fabricated on the low-cost FR-4 substrate (*ε_r_* = 4.4, tan *δ* = 0.02, thickness (*t*) = 1.6 mm) with dimensions *L*_1_ × *W*_1_ = 25 × 25 mm^2^. The antenna consists of a G-shaped monopole radiator excited through a 50-Ω microstrip-line feed, and a modified rectangular-shaped ground plane formed at the bottom surface of the dielectric substrate. By adding a vertical line strip to the monopole element, the fundamental mode of the monopole antenna can be degenerated into two orthogonal modes of equal amplitude and quadrature phase difference. The vertical line strip also enhances the impedance bandwidth by increasing the path of the surface current. Furthermore, a circular arc is etched on the ground surface to improve impedance matching at higher frequencies. The antenna was simulated by using a 3-D electromagnetic simulator, the ANSYS HFSS^®^, and the parameters of the designed antenna are presented in Table 1.

#### 2.1.1. Design Process

**Figure 2:**
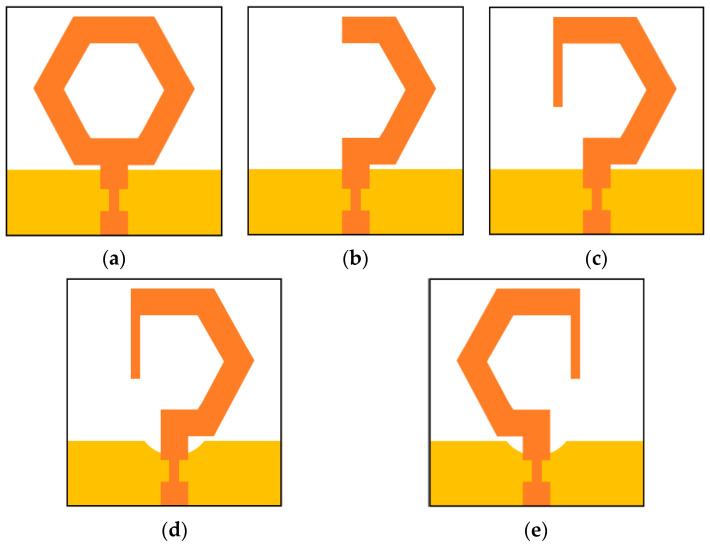
Evolution steps of the proposed CP monopole antenna: (**a**) antenna 1; (**b**) antenna 2; (**c**) antenna 3; (**d**) antenna 4; (**e**) mirror image of antenna 4.

The design procedure of the proposed monopole structure, which is used as an element of the presented MIMO antenna, is illustrated in Figure 2. A hexagon-shaped monopole resonator is fed by a microstrip feed line of 50 Ω. The proposed hexagonal monopole and the rectangular ground plane offer wide resonating bandwidth. The wideband behaviour can be obtained by circular, rectangular, or elliptical-shaped geometries, but the electrical size of the hexagon shape is larger, which results in longer surface current path, thereby covering the lower frequency range.

The central metal portion of the hexagon monopole radiator is etched out to form a hexagonal ring, which shows ultra-wide bandwidth (antenna 1) as displayed in Figure 2a. In the next step, a portion of the hexagonal ring is cut to form a C-shaped monopole (antenna 2) as illustrated in Figure 2b. The C-shaped monopole is open at one end. The vertical and horizontal arms of the C-shaped patch perturbs the current distribution of the radiator and generates electric fields (*E_x_* and *E_y_*) with unequal magnitudes. However, orthogonally directed horizontal and vertical electric fields of equal magnitude, with a 90° phase difference between them, can achieve the circular polarization operation.

In step-3, the length of the C-shaped radiator is varied, and a vertical line strip is integrated into the antenna 2 to balance horizontal and vertical electric fields and to introduce a 90° phase difference between them. With the addition of a straight line strip, the surface current length is increased, thus improving the impedance matching at lower frequencies. The CP antenna 3 is illustrated in Figure 2c. The introduced vertical line strip balances the magnitude ratio (*E_x_*/*E_y_*) and 90° phase difference between horizontal and vertical electric fields.

**Figure 3:**
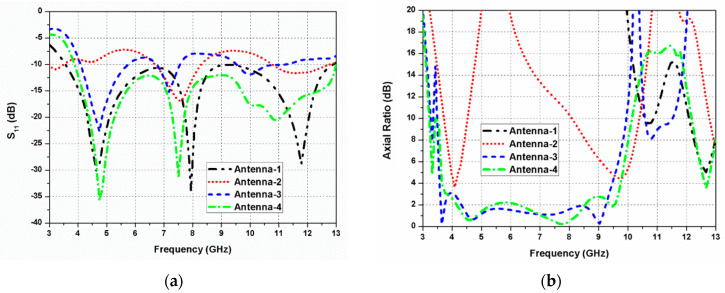
Simulated response of the evolution steps: (**a**) reflection coefficients; (**b**) axial ratio.

**Figure 4:**
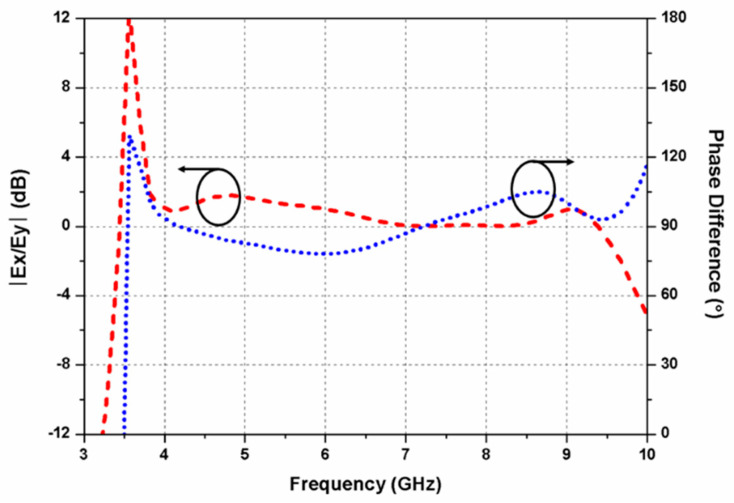
Simulated amplitude ratio and phase difference of the antenna element.

**Figure 5:**
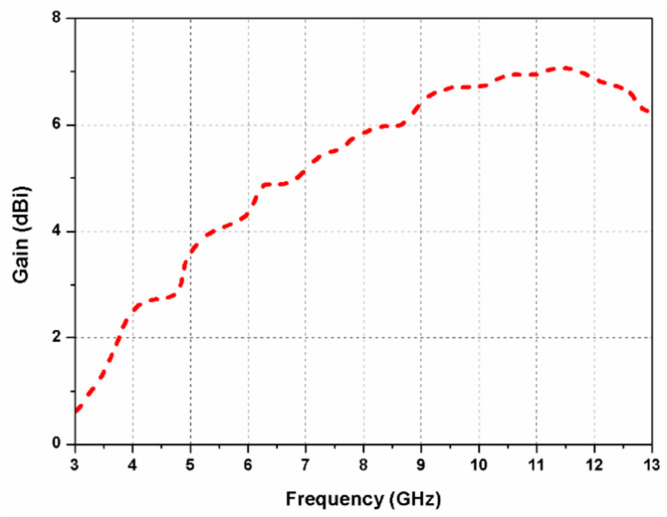
Simulated gain of the proposed antenna element.

Furthermore, as displayed in Figure 2d, an arc-shaped region is etched out from the rectangular ground plane beneath the radiator to improve impedance matching (antenna 4). The S_11_ and axial ratio curves of the antennas 1, 2, 3, and 4 are presented in Figure 3a,b, respectively. By etching out an arc-shaped region from the ground plane of antenna 3, the impedance matching improves significantly. It can be observed from Figure 3b that antennas 1 and 2 are LP, while antennas 3 and 4 are CP. The 3-dB ARBW of the proposed antenna 4 ranges from 3.8–9.7 GHz and impedance bandwidth (S_11_ ≤ −10 dB) from 3.8–13 GHz. In the proposed G-shaped antenna, the *E_x_*/*E_y_* ratio of two orthogonal transverse fields is closer to 0 dB with 90° phase difference between them within the C-band as displayed in Figure 4. The simulated gain of the proposed G-shaped antenna element is displayed in Figure 5.

In Figure 2e, a mirrored-shape of the designed monopole antenna element is illustrated, which is the mirror image of the monopole antenna (shown in Figure 1). The radiation performance of the designed antenna element and its mirror image element are similar due to their identical shape and size except their sense of polarization. The proposed monopole antenna displays RHCP operation, whereas its mirror image displays LHCP operation.

**Figure 6:**
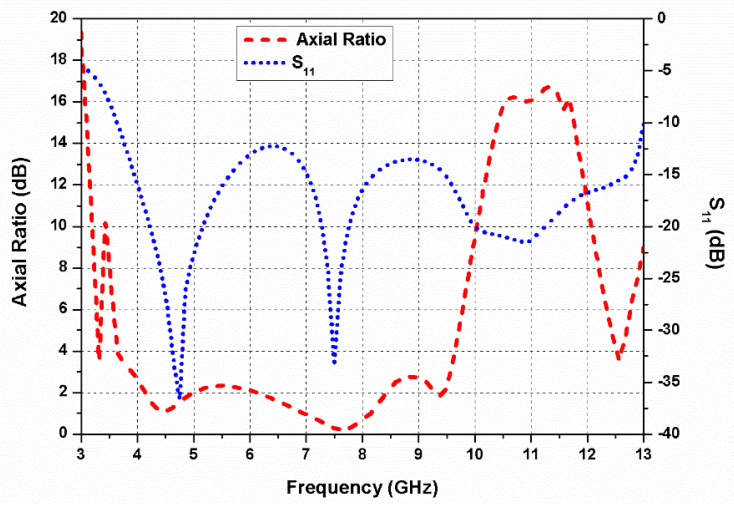
Simulated reflection coefficients and axial ratio of the mirrored-shape monopole antenna element.

Figure 6 shows the simulated S_11_ and axial ratio performance of the mirrored-shape monopole antenna. It is evident from the figure that the ARBW and impedance bandwidth of the mirrored-shape antenna are identical to the proposed antenna. The 3 dB ARBW ranges from 3.8–9.7 GHz, and impedance bandwidth (S_11_ ≤ −10 dB) varies from 3.8–13 GHz.

#### 2.1.2. Circular Polarization Mechanism

**Figure 7:**
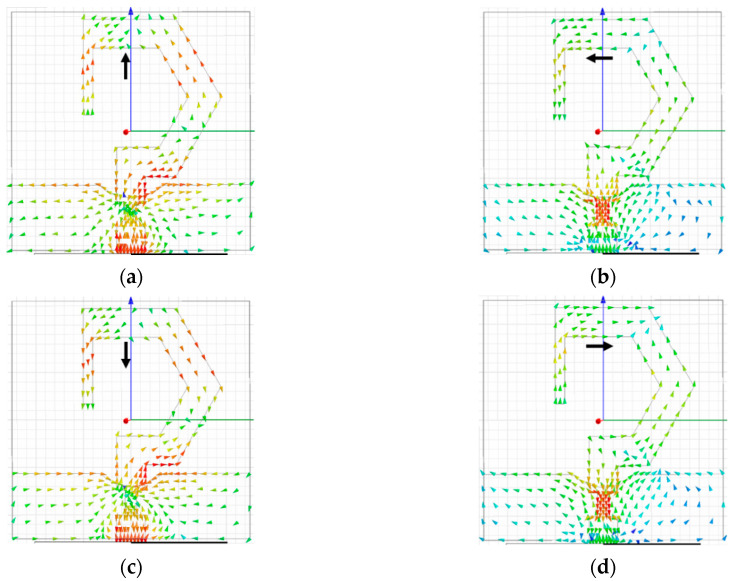
Surface current distribution at 6 GHz: (**a**) *ωt* = 0°; (**b**) *ωt* = 90°; (**c**) *ωt* = 180°; (**d**) *ωt* = 270°.

The surface current distribution of the proposed monopole antenna is studied for explaining the circular polarization mechanism. Circular polarization can be achieved using two orthogonal resonant modes with equal amplitude and 90° phase variance between them. Figure 7a–d display the current distributions of the antenna element at 6 GHz (for 0°, 90°, 180°, and 270° phases, respectively). The current distribution changes with the variation in the feeding phase from 0° to 270°.

At 0° phase, the dominant current vectors are in +*y*-direction, whereas, at 180° phase, the dominant current vectors are in the −*y*-direction. At 90° and 270° phases, the current distribution is equal in magnitude and opposite in phase. Thus, the current vectors (varying with time) are rotating in a counter-clockwise manner, which illustrates the RHCP operation of the antenna.

#### 2.1.3. Circular Polarization Mechanism of the Mirrored-Shape Monopole Antenna

**Figure 8:**
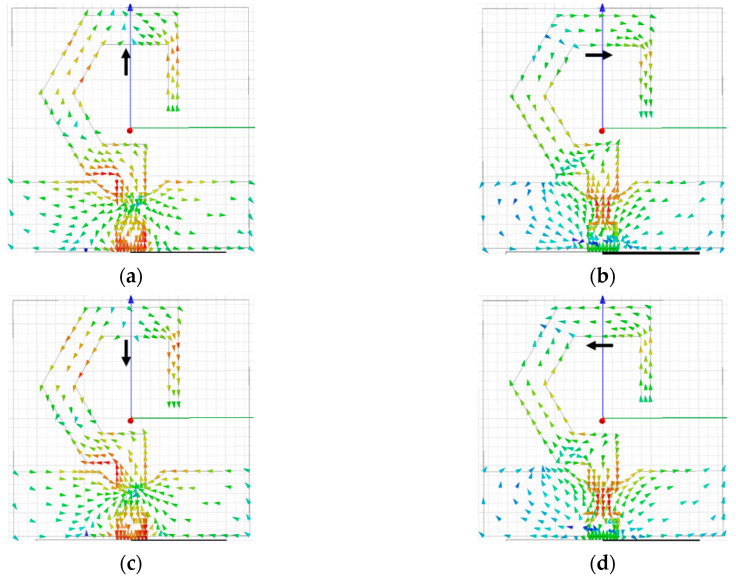
Surface current distribution at 6 GHz: (**a**) *ωt* = 0°; (**b**) *ωt* = 90°; (**c**) *ωt* = 180°; (**d**) *ωt* = 270°.

The surface current distribution of the mirrored-shape monopole antenna at 6 GHz (at four different time instants (*ωt*): 0°, 90°, 180°, and 270°) is shown in Figure 8a–d, respectively. The current distribution changes with the variation in the feeding phase from 0° to 270°. At 0° phase, the current vectors are predominantly along the +*y*-direction, and changes to the +*x*-direction at 90° phase.

On changing the feeding phases to 180° and 270°, the direction of the current vectors becomes opposite to 0° and 90°, respectively. It is noted that the surface current rotates in a clockwise manner, when observed from the +*z*-direction, thus validating the LHCP operation of the mirror image antenna element.

#### 2.1.4. Parametric Analysis

To obtain the optimized dimensions of the proposed G-shaped antenna, one of the design parameters is varied while other parameters are kept constant. The ARBW and impedance bandwidth of the antenna element can be enhanced by tuning the length (*h*_5_) of the straight line strip, and gap (*h*_6_) between the monopole radiator and the ground plane.

**Figure 9:**
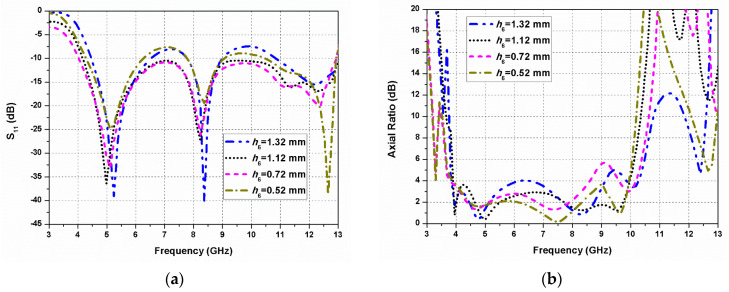
Effect of *h*_6_ on the antenna performance: (**a**) S_11_; (**b**) axial ratio.

The S_11_ and axial ratio variations as a function of the gap (*h*_6_) between the G-shaped radiating patch and the ground plane are shown in Figure 9a,b, respectively. It is evident that *h*_6_ affects the 3-dB ARBW significantly, but affects the impedance bandwidth relatively less. In fact, the spacing *h*_6_ can influence the relative position of the radiating patch in the *y*-direction. Therefore, the circular polarization behaviour is controlled by the coupling between the ground plane and the radiator. It can be noticed from Figure 9b that the 3-dB ARBW is decreasing for larger *h*_6_.

**Figure 10:**
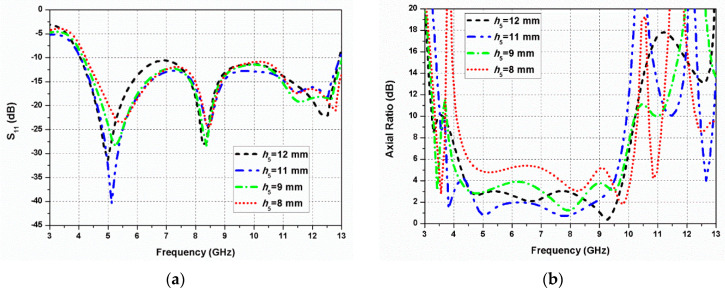
Effect of *h*_5_ on the antenna performance: (**a**) S_11_; (**b**) axial ratio.

Figure 10 presents the S_11_ and axial ratio variations of the length (*h*_5_) of the straight line strip. It can be observed from Figure 10a that the variation of *h*_5_ has a small effect on the impedance bandwidth, but a significant effect on the ARBW as illustrated in Figure 10b. As the length of the vertical line strip is increased, the 3-dB ARBW is increased. The length *h*_5_ in fact affects the (*E_x_*/*E_y_*) ratio and phase difference between the electric fields, and the ARBW of the antenna can be optimized by adjusting *h*_5_.

### 2.2. Four-Element Circularly Polarized Multiple-Input–Multiple-Output (CP MIMO) Antenna Design

**Figure 11:**
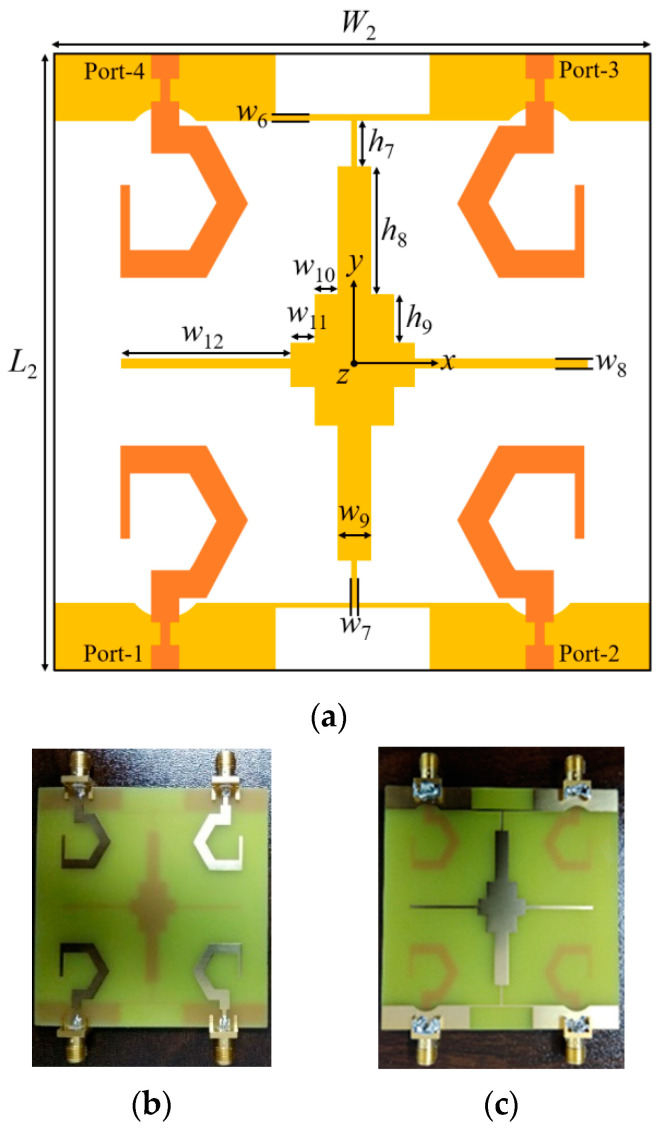
Proposed dual-circularly polarized multiple-input–multiple-output (CP MIMO) antenna: (**a**) schematic; (**b**) photograph of the top surface; (**c**) photograph of the bottom surface.

Utilizing the monopole antenna element, the proposed four-element dual-CP MIMO/diversity antenna layout is illustrated in Figure 11. The antenna consists of four identical G-shaped monopole elements, which are organized in a mirror-image pattern to each other. This mirrored pattern of the G-shaped elements supports polarization diversity. Due to this arrangement, the antenna radiates RHCP waves when the elements 1 and 3 are excited, and LHCP waves when the elements 2 and 4 are excited. An I-shaped strip is used to connect the ground planes of the G-shaped elements as shown in Figure 11a. Further, for obtaining improved isolation among the antenna elements, a cross-shaped structure is incorporated in the ground plane of the dual-CP MIMO antenna. The pictures of the dual-CP MIMO antenna prototype (top and bottom views) are shown in Figure 11b,c, respectively. The MIMO antenna is fabricated on the FR-4 substrate of dimension 70 × 68 × 1.6 mm^3^. SMA connectors are used for experimental measurements and validation of the simulated results. The parameters of the proposed dual-CP MIMO antenna are listed in Table 1.

#### Design Process

**Figure 12:**
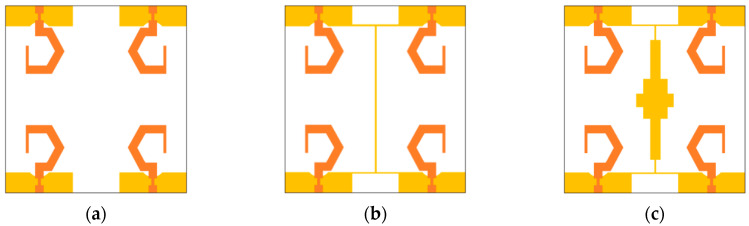
Dual-CP MIMO antenna: (**a**) MIMO antenna 1; (**b**) MIMO antenna 2; (**c**) MIMO antenna 3.

The design steps of the proposed dual-CP MIMO antenna are illustrated in Figure 12a–c. In the present work, the primary goal is to achieve a wide ARBW with connected ground planes of the G-shaped antenna elements. However, it is hard to hold wide ARBW of the radiating elements when they are closely located with connected ground planes. Initially, four G-shaped radiating elements are arranged in a mirrored-image pattern (MIMO antenna 1), as shown in Figure 12a. In typical MIMO antennas, the inter-element coupling can be suppressed by introducing enough spacing among the radiating patches. Therefore, in the proposed dual-CP MIMO antenna, proper spacing is introduced between the patches to restore ARBW of the G-shaped elements.

An I-shaped strip is implanted at the centre of the designed antenna to connect the ground planes of the G-shaped resonating elements (MIMO antenna 2) as displayed in Figure 12b. It is noticed that the ARBW of the antenna deteriorates significantly by introducing the I-shaped strip between the antenna elements. Larger isolation or minimum coupling is required among the G-shaped elements to improve the ARBW of the resonating element. In addition, isolation among the MIMO antenna ports can be enhanced further using decoupling elements. Therefore, a decoupling element is incorporated at the center of the antenna (MIMO antenna 3) as displayed in Figure 12c. With this extra decoupling strip, the ARBW of the designed antenna increases considerably. The position of the decoupling element is adjusted in such a way that the minimum coupling between the resonating elements and wide ARBW can be achieved.

**Figure 13:**
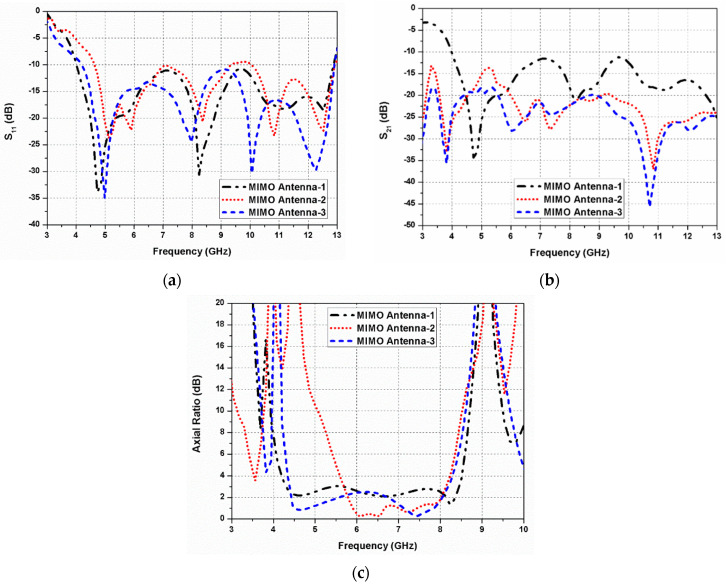
Simulated response of the design steps: (**a**) reflection coefficients; (**b**) transmission coefficients; (**c**) axial ratio.

The S_11_, S_21_, and axial ratio variations of the MIMO antenna 1, MIMO antenna 2, and MIMO antenna 3 are presented in Figure 13a–c, respectively. It is observed that the decoupling element has a minor effect on the impedance bandwidth, but significantly influences the 3-dB ARBW and isolation. The circular polarization behaviour is affected due to the coupling between the G-shaped radiators, and it can be improved by introducing space and decoupling elements between the resonating elements.

The final proposed MIMO/diversity antenna layout, with connected ground planes of the G-shaped radiators, is displayed in Figure 11a. The rectangular arm (introduced horizontally in the proposed MIMO antenna) of the decoupling element further decreases coupling between the G-shaped elements (1 and 4/2 and 3). In addition to isolation enhancement, the decoupling element connects the ground planes of all the G-shaped antenna elements, and, therefore, it is located at the bottom surface of the dielectric substrate.

## 3. Results Discussion

**Figure 14:**
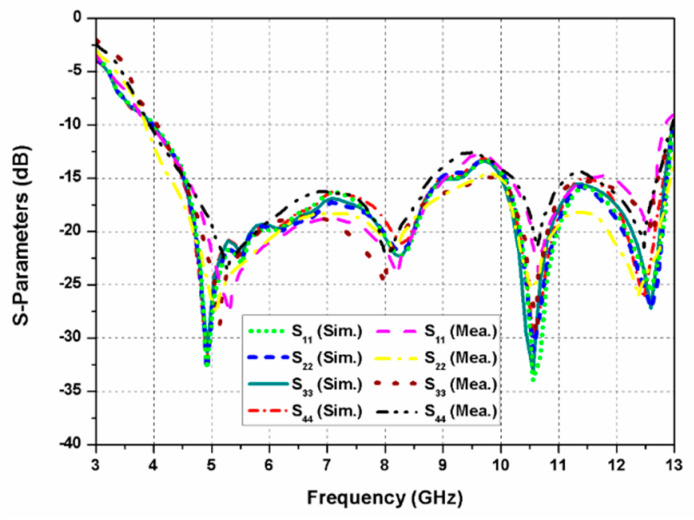
Reflection coefficients of the proposed dual-CP MIMO antenna.

The simulated and measured reflection coefficients of the proposed dual-CP MIMO antenna are presented in Figure 14. Owing to the identical resonating elements, the S-parameters (S_11_, S_22_, S_33_, and S_44_) at all four ports are almost similar. The impedance bandwidth (S_11_ ≤ −10 dB) of the MIMO antenna is 105.9% (4–13 GHz).

**Figure 15:**
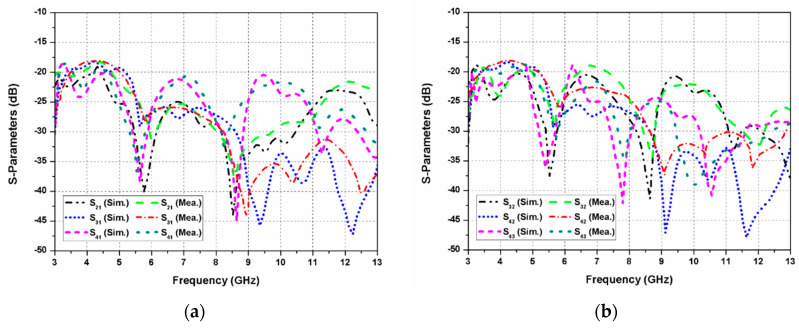
Transmission coefficients of the proposed dual-CP MIMO antenna: (**a**) with reference to port-1; (**b**) other ports.

The simulated and measured transmission coefficients (S_21_, S_31_, S_41_, and S_32_, S_42_, S_43_) of the proposed dual-CP MIMO antenna are presented in Figure 15a,b, respectively. It can be noticed that isolation greater than 18 dB is offered between the resonating G-shaped elements. During the measurement, one element of the proposed antenna is excited, while other elements are matched with the 50 Ω loads.

**Figure 16:**
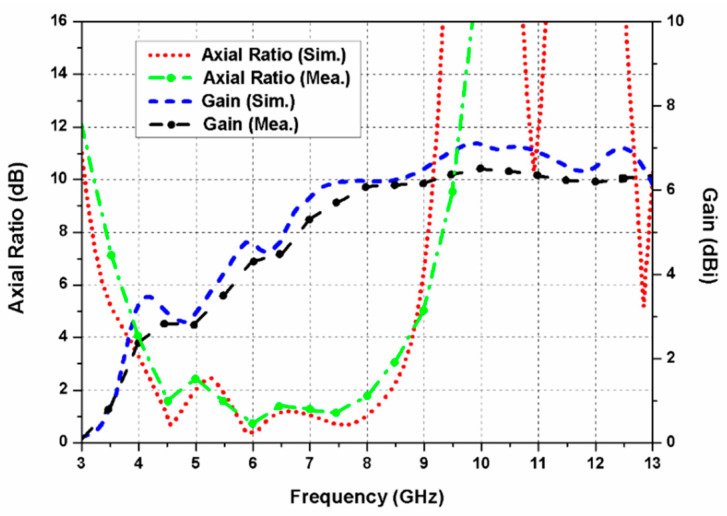
Axial ratio and gain of the proposed dual-CP MIMO antenna.

The simulated and measured axial ratio of the proposed dual-CP antenna is displayed in Figure 16. It can be observed from the figure that the antenna possesses a very wide 3 dB ARBW of 67.7% (4.2–8.5 GHz) with 100% bandwidth overlap between the axial ratio and impedance bandwidths.

The simulated and measured gain curves of the proposed dual-CP MIMO antenna are depicted in Figure 16. The measured gain ranges from 2.5 to 6.4 dBi in the resonating range (4–13 GHz) of the antenna. It is observed that the simulated and measured outcomes agree closely with each other. Small differences between them are mainly due to fabrication and soldering errors.

**Figure 17:**
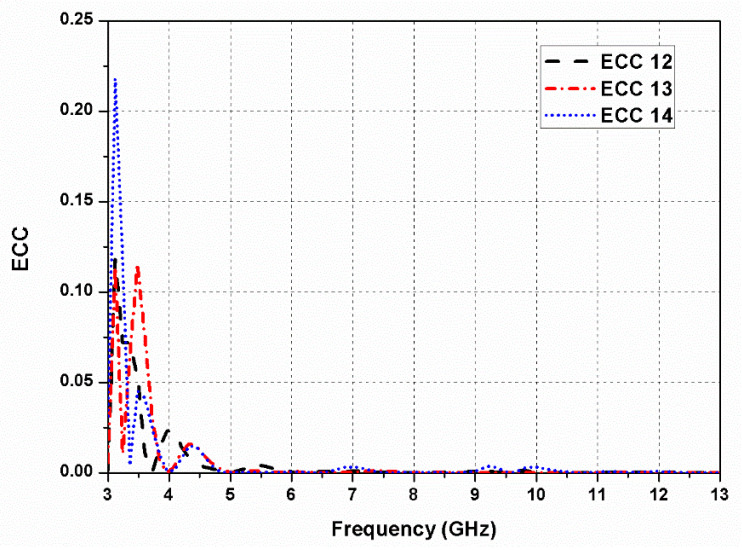
Envelope correlation coefficient (ECC) of the proposed dual-CP MIMO antenna.

The envelope correlation coefficient (ECC) is used to validate the performance of the designed antenna for diversity applications. It is used to evaluate the influence of different propagation paths taken by the radio frequency (RF) signal to reach the receiving antenna. In other words, it can calculate correlation or isolation between different channels in the MIMO system. The ECC value of the MIMO antenna should lie in the range of 0–1. For a multi-antenna system, the ECC is expressed as [27]:

(1) $$\rho_{e}=\frac{{\left|\int \int \left[\vec{E_{1}}\left(\theta ,\;\varphi \right)\vec{E_{2}}\left(\theta ,\;\varphi \right)\right]\;d\Omega \right|}^{2}}{\int \int {\left|\vec{E_{1}}\left(\theta ,\;\varphi \right)\right|}^{2}d\Omega \int \int {\left|\vec{E_{2}}\left(\theta ,\;\varphi \right)\right|}^{2}d\Omega }$$

where Ω, *φ*, and *θ* are the respective solid, azimuthal, and elevation angles, and *E* is the radiated field pattern. Figure 17 presents the diversity characterization of the dual-CP MIMO antenna design, and it can be noticed that ECC is smaller than 0.25 in the whole resonating band.

**Figure 18:**
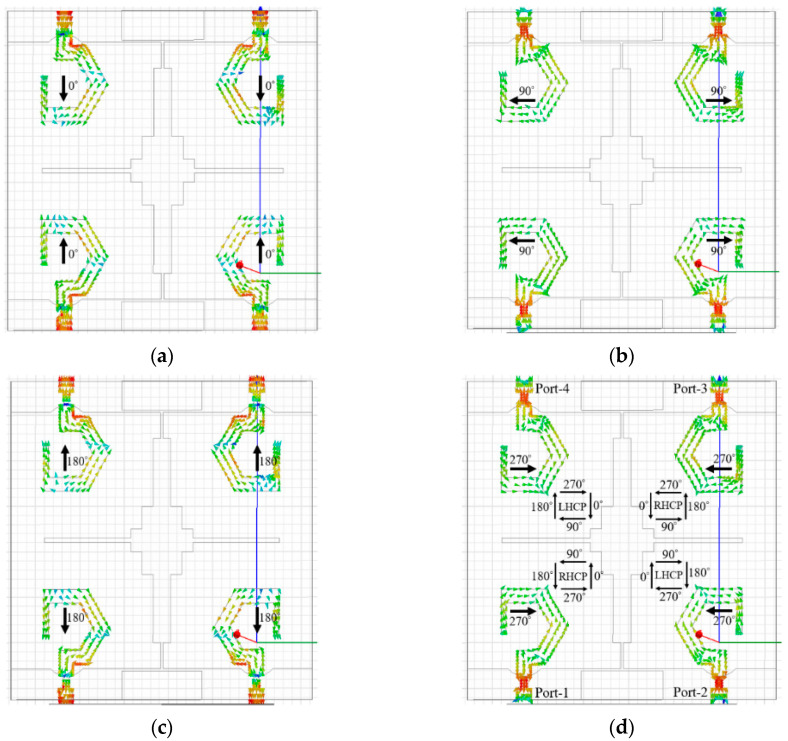
Vector current distribution at 6 GHz: (**a**) *ωt* = 0°; (**b**) *ωt* = 90°; (**c**) *ωt* = 180°; (**d**) *ωt* = 270°.

The surface current distributions are further used to understand the circular polarization mechanism of the proposed MIMO antenna. Figure 18a–d shows the current distributions of the antenna element at 6 GHz (for 0°, 90°, 180°, and 270° phases, respectively) when all four ports are excited simultaneously.

At 0° phase, the dominant current vectors (at port-1 and port-2) are in the +*y*-direction, whereas, at the 180° phase, the dominant current vectors (at port-1 and port-2) are in the −*y*-direction. At 90° and 270° phases, the current distributions at port-1 and port-2 are equal in magnitude and opposite in phase. Likewise, at 0° phase, the dominant current vectors (at port-3 and port-4) are in the −*y*-direction, whereas, at 180° phase, the dominant current vectors (at port-3 and port-4) are in the +*y*-direction. At 90° and 270° phases, the current distributions at port-3 and port-4 are equal in magnitude and opposite in phase. Consequently, as shown in Figure 18d, the current vectors at port-1 and port-3 are rotating in a counter-clockwise direction, illustrating RHCP operation of the antenna elements 1 and 3. In addition, the current vectors at port-2 and port-4 are rotating in a clockwise direction, showing LHCP operation of the antenna elements 2 and 4.

**Figure 19:**
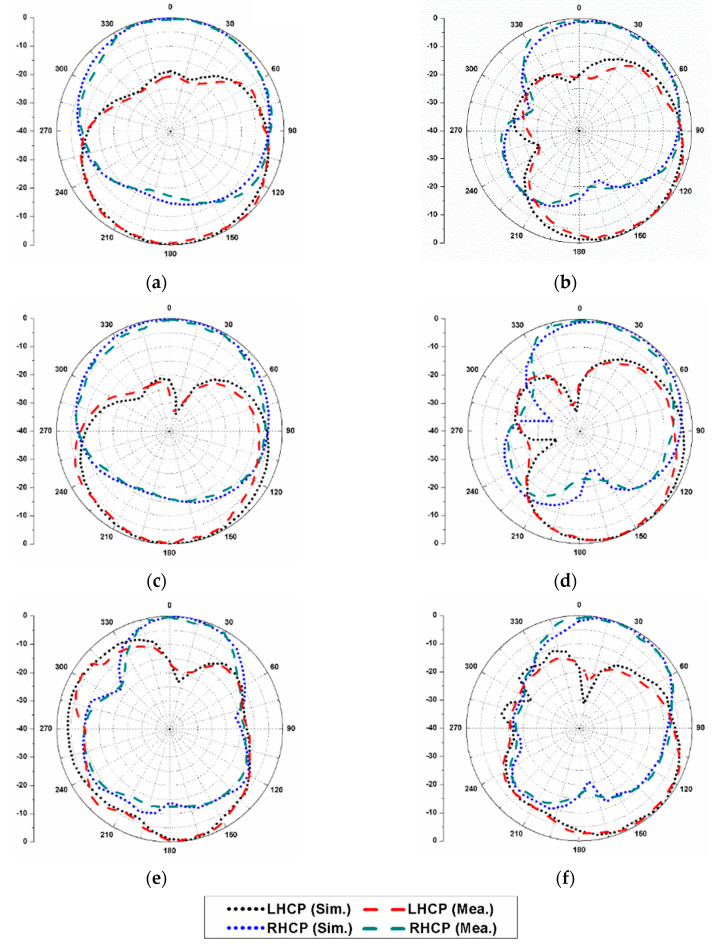
Radiation patterns of the proposed dual-CP MIMO antenna (when port-1 is excited): (**a**) 5 GHz, *φ* = 0°; (**b**) 5 GHz, *φ* = 90°; (**c**) 6.5 GHz, *φ* = 0°; (**d**) 6.5 GHz, *φ* = 90°; (**e**) 8 GHz, *φ* = 0°; (**f**) 8 GHz, *φ* = 90°.

**Figure 20:**
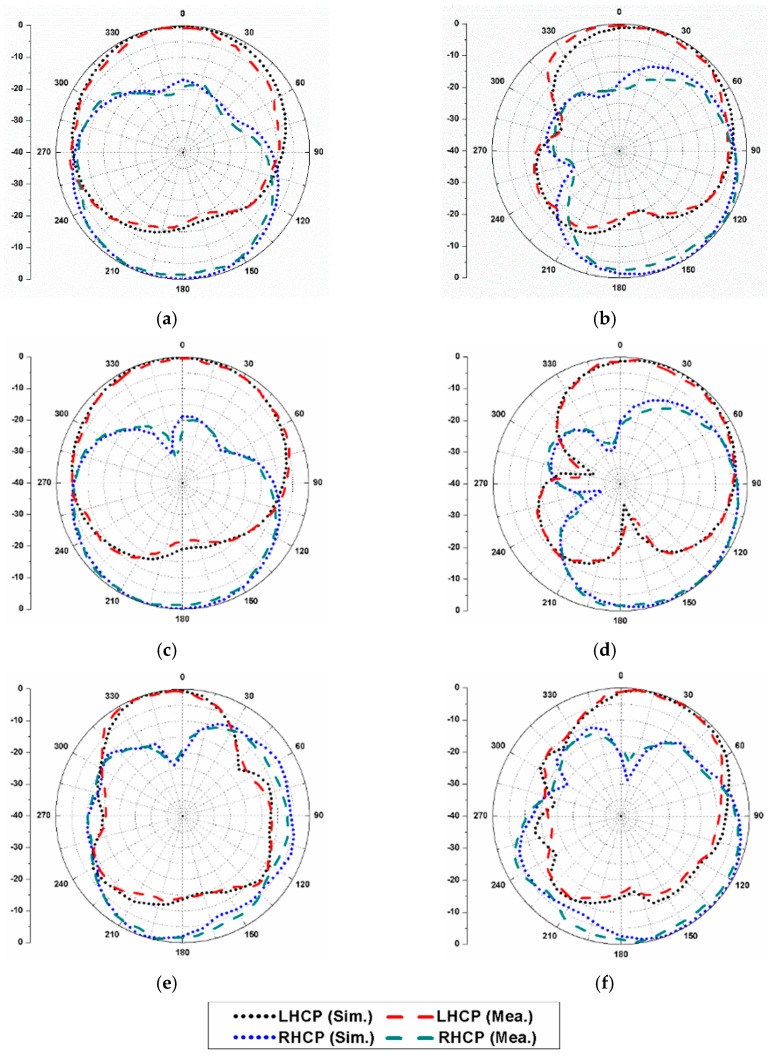
Radiation patterns of the proposed dual-CP MIMO antenna (when port-2 is excited): (**a**) 5 GHz, *φ* = 0°; (**b**) 5 GHz, *φ* = 90°; (**c**) 6.5 GHz, *φ* = 0°; (**d**) 6.5 GHz, *φ* = 90°; (**e**) 8 GHz, *φ* = 0°; (**f**) 8 GHz, *φ* = 90°.

The simulated and measured radiation patterns of the proposed dual-CP antenna are displayed in Figure 19 at 5 GHz, 6.5 GHz, and 8 GHz when port-1 is excited. It can be noticed that in both the planes (*φ* = 0°, 90°), the antenna demonstrates RHCP operation in the +*z*-direction. Similarly, Figure 20 displays the simulated and measured radiation patterns at 5 GHz, 6.5 GHz, and 8 GHz when port-2 is excited. It can be noticed that the antenna operates in LHCP radiation in both planes (*φ* = 0°, 90°). Therefore, the antenna radiates RHCP waves when the elements 1 and 3 are excited, and LHCP waves when the elements 2 and 4 are excited. This validates polarization diversity performance of the presented MIMO antenna.

Table 2 compares the behaviour of the proposed dual-CP MIMO antenna with other antennas reported in the literature. The CP MIMO antennas presented in [17,18] could generate only RHCP waves. The MIMO antennas in [19,21,23,25] featured dual circular polarization, but their axial ratio and impedance bandwidths were quite small. The antenna in [20] provided wider ARBW, but its ground planes were not connected to each other, and therefore it could not be used in practical applications. In addition, the antennas presented in [22,24] had a larger size and separated ground planes. As compared to the antennas in [17,18,19,20,21,22,23,24,25], the proposed dual-CP four-element MIMO antenna offers broader axial ratio and impedance bandwidths and relatively small size. It contains four resonating elements that offer extra degrees of freedom to increase link reliability and communication capacity. A high isolation is achieved with very small element separation distance. Also, an equal voltage level is provided in the ground surface of the proposed antenna by using an I-shaped strip, which makes it practical for C-band applications.

## 4. Conclusions

This paper presents a compact dual-CP MIMO antenna with broad ARBW. Each antenna element consists of a microstrip line-fed G-shaped monopole radiator, and a rectangular ground plane with an arc-shaped region etched out from it. The proposed MIMO geometry consists of four G-shaped elements arranged in a mirrored pattern to each other. The proposed MIMO/diversity antenna radiates RHCP waves when the radiators 1 and 3 are excited, and LHCP waves when the radiators 2 and 4 are excited. The antenna configuration is fabricated, and measured results show in close agreement with the simulated results. Also, the antenna possesses a very wide impedance bandwidth of 105.9% (4–13 GHz) and 3 dB ARBW of 67.7% (4.2–8.5 GHz). It shows dual circular polarization characteristics with a peak gain of 6.4 dBi. These properties make the presented antenna a good candidate for various C-band wireless communication applications.

## Figures and Tables

**Table 1:** Design details of the proposed antenna.

Dimensions	Value (mm)	Dimensions	Value (mm)
*L*_1_	25	*r*_3_	4.5
*W*_1_	25	*t*	1.6
*h*_1_	2.5	*L*_2_	70
*h*_2_	2.6	W_2_	68
*h*_3_	5.7	*h*_7_	5.65
*h*_4_	6.85	*h*_8_	15
*h*_5_	10	*h*_9_	5
*h*_6_	0.92	*w*_6_	0.5
*w*_1_	3	*w*_7_	0.5
*w*_2_	1.2	*w*_8_	1
*w*_3_	4.5	*w*_9_	4
*w*_4_	9.75	*w*_10_	2.5
*w*_5_	1	*w*_11_	2.5
*r*_1_	9.5	*w*_12_	19.5
*r*_2_	6		

**Table 2:** Comparison of the proposed dual-CP MIMO antenna with other CP MIMO/diversity antennas.

Ref.	No. of Ports	Antenna Size (mm^3^)	(S_11_ ≤ −10 dB) Band (GHz)	Impedance BW (GHz)/%	3-dB Axial Ratio Band (GHz)	3-dB ARBW (GHz)/%	Polarization	ECC	Isolation (dB)	Peak Gain (dB)	Connected Ground
[17]	2	25 × 30 × 1.524	5.4–6.1	0.7/12.2	5.8, 5.9, 6	—	RHCP	<0.06	>13	4.3	No
[18]	2	29 × 48 × 1.6	5.4–6.2	0.8/13.8	5.61–5.7	0.09/1.6	RHCP	<0.01	>15	4	Yes
[19]	2	60 × 60 × 1.6	2–4.76	2.76/81.6	2–3.7	1.7/59.6	LHCP/RHCP	—	>15	4	Yes
[20]	2	32 × 32 × 1	1.4–8.73	7.33/144.7	3.74–8.8	5.06/81.1	LHCP/RHCP	<0.5	>20	3.8	No
[21]	2	100 × 150 × 0.8	2.47–2.55	0.08/3.2	2.5–2.66	0.16/6.2	LHCP/RHCP	<0.003	>20	6.1	Yes
[22]	2	66 × 66	1.82–2.57	0.75/34.2	2.15–2.6	0.45/18.9	—	<0.01	>24	4	No
[23]	2	13.7 × 36.2 × 0.813	5.2–6.3	1.1/19.1	5.2–6.3	1.1/19.1	LHCP/RHCP	<0.002	>22	5.8	No
[24]	4	165 × 165 × 1.6	1.71–1.88, 3.3–3.7, 5.12–5.37	0.17/9.5, 0.4/11.4, 0.25/4.8	3.56–3.67, 5.16–5.29	0.11/3, 0.13/2.5	—	—	>37	5	No
[25]	4	27.69 × 97 × 1.524	5.49–6.024	0.534/9.3	5.744–5.83	0.086/1.5	LHCP/RHCP	<0.15	>33	5.34	No
Prop.	4	70 × 68×1.6	4–13	9/105.9	4.2–8.5	4.3/67.7	LHCP/RHCP	<0.25	>18	6.4	Yes
